# Paltusotine: The first FDA approved oral treatment for acromegaly

**DOI:** 10.1097/MS9.0000000000004781

**Published:** 2026-02-12

**Authors:** Bisma Faraz, Sania Sheikh, Muhammad Areeb Ul Haq, Ehtisham Haider, Muhammad Umer Farooq Mujahid, Sakarie Mustafe Hidig

**Affiliations:** a Department of Medicine, Karachi Medical and Dental College, Pakistan; b Department of Medicine, Allama Iqbal Medical College, Lahore, Pakistan; c Department of Medicine, Punjab Medical College, Faisalabad, Pakistan; d Department of Medicine, University of Health Sciences, Lahore, Pakistan; e Research Center, Hargeisa Group Hospital, Hargeisa, Somaliland, Somalia

Paltusotine is a non-peptide, orally active selective agonist of somatostatin receptor-2 that regulates insulin-like growth factor-1 (IGF-I) levels, which are high in individuals with acromegaly. Acromegaly is a chronic condition resulting from over-secretion of growth hormone (GH) – somatostatin – and its secondary mediator, IGF-I and is most commonly caused by a pituitary adenoma. The prevalence rate of the condition is estimated to be 5.9 per 100 000 in the general population and the yearly incidence of the condition stands at 0.38 per 100 000 people^[1]^. The initial therapy of the GH-secreting pituitary tumor is usually surgery; however, nearly 45% of the patients failed to obtain sufficient biochemical control, which is a reduction in GH concentrations and normalization of IGF-I. Acromegaly can be treated through surgical excision of the pituitary tumor and/or administration of lanreotide or octreotide injections every 4 weeks to regulate IGF-1 levels. Injection site irritation is a frequently reported side effect of various injectable therapies^[2]^. The preparation of this letter has been done in accordance with Titan 2025^[3]^.

In patients who remain uncontrolled following surgery or in patients who are not suitable to be operated on, medical treatment is advised. The current treatment is the injectable somatostatin receptor ligands (SRLs). These act to reduce GH secretion by acting on somatostatin receptor subtypes, particularly, SSTR2. Injectable SRLs are effective but are problematic in practice and clinically. Local side effects as a result of monthly depot injections may include pain, nodules, bruising, fibrosis, and inflammation. They also come with a treatment burden that may diminish adherence and general quality of life^[4]^. In clinical terms, the restrictions can be translated to less than ideal disease control in practice, which means that more convenient and tolerable variants should be sought. To improve and solve this paradigm, paltusotine has recently received regulatory approval by the Food and Drug Administration in United States. It is the first once-daily, orally bioavailable, non-peptide SSTR2 agonist for adults with acromegaly who did not respond adequately to surgery or cannot undergo surgery. Its efficient gastrointestinal absorption represents a notable step forward in the development of oral SRLs^[4,5]^.

The safety concerns and adverse effects of paltusotine were investigated in PATHFNDR-1 and PATHFNDR-2 trials. It is overall well-tolerated; however, the most common side effects of administering paltusotine are of mild-to-moderate extent and coincide with the previously known class effects of SRLs or symptoms of acromegaly underlying. These comprise of gastrointestinal problems like diarrhea, stomach pain, nausea, decreased appetite, dyspepsia, hyperglycemia, and cholelithiasis, which are usually brief and arise at an early part of treatment, whereas, headache, arthralgia, fatigue, and hyperhidrosis usually have something to do with the illness as opposed to the medication. The volume of pituitary tumor was also stable or decreasing in the case of the treated patients, which means that the issues related to tumor progression are not to worry about and the good safety characteristic. The co-administration of strong CYP3A4 inducer and proton pump inhibitor reduced exposure of paltusotine. Further trails should focus on long-term implications of paltusotine administration^[6]^.

To address these limitations, research has increasingly focused on the development of oral SRLs. A recent systematic review emphasized on the problems of developing orally administered SRLs, including low intestinal permeability and degradation in the acidic gut environment, and new methods of overcoming these obstacles, including permeability enhancers, enteric coatings, and absorption enhancers^[1,7]^.

Paltusotine is more expensive than fg-SRLs of standard of care, which represents a considerable increase in pharmacologic cost; however, there is impressive convenience and patient-focused benefits with paltusotine, which justify the premium-priced therapy. It is restricted to adults who have acromegaly following inappropriate response to surgery or ineligible to surgery. Contraindicated in children, first-line choice in all situations, and in other disorders, although is being investigated in carcinoid syndrome^[5]^.

Two phase 3 randomized trials, double-blind, placebo-controlled trials were in favor of its approval. Patients receiving continuous injectable paltusotine or placebo in the PATHFNDR-1 trial had been previously receiving stable injectable monotherapy with SRL at 1.0xl in ULN excluded groups and subjected to 36 weeks of therapy with a monitored IGF-1 level (<1.0xl) before the switch. In weeks 34–36, 83.3% of the patients who were on paltusotine and 3.6% on placebo met the major outcome, which was maintenance within the range of IGF-1 of less than 1.0 × ULN. In the paltusotine group, there was no significant change in IGF-1 because it ranged 0.83–0.89x ULN, whereas, in the placebo group, it rose significantly 0.82–1.68x ULN^[2]^. In PATHFNDR-2, IGF-1 normalization was observed in 42.5 and 2.4% of patients treating naive and treated randomly to paltusotine or a placebo in 24 weeks, respectively^[6]^.

The randomized periods are short (24–36 weeks), and studies are being conducted to investigate the long-term durability of biochemical control, its effect on tumor volume, and potential delayed adverse events^[2]^. Moreover, trial populations may have more favorable tumor biology (e.g., densely granulated adenomas), which raises questions regarding the efficacy of the drug in more resistant morphologies (e.g., sparsely granulated adenomas). Adherence issues may also arise from the need for a daily oral dosage, particularly for those who are already taking many drugs^[5,6]^.

Because of this, there is no current information on the long-term effectiveness of paltusotine. To test both safety and effectiveness, the open-label phase II ACROBAT Advance study (NCT04261712) is in progress and will conduct the therapy over a period of 1 year^[7]^. Besides the short-term goals, trials in future must explore the long-term response, effects on tumor volume, and patient outcomes in terms of quality of life. A head-to-head comparison of oral therapy using injectable SRLs and cost effectiveness studies would also be insightful on clinical and cost benefits of oral therapy.

Therefore, paltusotine is a new kind of medication to be used in the first-line medical intervention, alongside oral octreotide and the new long-acting subcutaneous octreotide, which should offer a wider range of alternatives to match the preferences of the patient, but remain effective. It is pertinent to mention that an activity as effective as the current first-line medical regimens is suggested that around half of the patients are going to need extra interventions other than these SRLs, and these medications must not substitute other medication techniques, including the incorporation of cabergoline or a switch to pegvisomant or pasireotide to patients with an ineffective response^[8,9]^.

Paltusotine is a pioneer in managing acromegaly since it is the first oral and non-peptide SSTR2 agonist and a replacement of injectable SRLs. Its positive tolerability and efficacy open the path to more convenient care of patients. It also equips the physician with the personalized treatment strategies in patients with acromegaly, thus improving the overall clinical decision-making. The paltusotine is also being considered for the carcinoid syndrome^[10]^. Oral SRLs, such as paltusotine, have the potential to change the quality of medical treatment of acromegaly by offering an efficient, patient-friendly, and long-term mechanism of control over the disease should the long-term outcomes confirm such effects.

## Data Availability

My manuscript has no associated data.
